# Role of Institutionalization in Interoception, Emotion Regulation, and Prosocial Behavior in Preschool Children

**DOI:** 10.3390/brainsci16060630

**Published:** 2026-06-12

**Authors:** Zamara Cuadros, María José Escobar-Falla, Marisol Correa, Eduar Herrera

**Affiliations:** Departamento de Estudios Psicológicos, Universidad Icesi, Cali 760031, Colombia; izcuadros@icesi.edu.co (Z.C.); maria.escobar10@u.icesi.edu.co (M.J.E.-F.); marisol.correa@u.icesi.edu.co (M.C.)

**Keywords:** interoception, institutionalization, emotion regulation, prosocial behavior, preschool children

## Abstract

**Highlights:**

**What are the main findings?**

- Institutionalized children were more likely to underestimate internal bodily signals.
- Non-institutionalized children showed greater social cooperation.

**What are the implications of the main findings?**

- Early institutionalization may affect interoceptive and socio-emotional development.
- Early interventions may enhance emotion regulation and prosocial functioning.

**Abstract:**

**Background/Objectives:** Although early institutionalization has been linked to socioemotional difficulties, its relationship with interoception in early childhood remains unclear. This study examined differences in interoception, emotion regulation, and prosocial behavior between institutionalized preschool children (IPC) and noninstitutionalized preschool children (NIPC) and explored the associations among these domains. **Methods:** In total, 51 children aged 4–6 years (26 IPC, 25 NIPC) participated in this study. Interoceptive accuracy (IAc) was assessed using an adapted Jumping Jack Paradigm that combined subjective reports and objective heart rate measures. Interoceptive sensitivity was evaluated using the iBEAT task based on gaze duration toward synchronous and asynchronous stimuli. Cooperation was measured using a joint fishing task, and emotion regulation was assessed using a delayed gratification task and the Early Emotion Regulation Behavior Questionnaire. Group differences were analyzed using one-way analysis of variance. Regression analyses were performed to explore the associations among variables. **Results:** Both groups had IAc values close to zero, indicating overall correspondence between subjective and objective signals. However, IPC showed more negative values, indicating underestimation, whereas NIPC showed more positive values, indicating overestimation. No significant differences in interoceptive sensitivity were found, and no evidence of discrimination between synchronous and asynchronous stimuli emerged. Compared with the IPC, the NIPC exhibited greater cooperation. No group differences were found in inhibitory control, although differences were observed in specific emotion regulation strategies. Regression analyses indicated that institutionalization and interoceptive sensitivity predicted IAc, whereas emotion regulation strategies and synchronous preference predicted cooperation. **Conclusions:** The results suggest that early institutionalization may induce changes in interoception, emotion regulation, and cooperation.

## 1. Introduction

Institutionalization has been consistently associated with disruptions in social functioning during childhood and adolescence [1,2,3,4,5,6]. This association is particularly evident in the domain of emotional self-regulation. Evidence indicates that cumulative childhood trauma, including emotional, physical, and sexual abuse, predicts both externalizing and internalizing symptoms in institutionalized children aged 10–18 years, primarily through maladaptive emotion regulation strategies [7]. Younger children, specifically those aged 4–6 years, who are exposed to institutional care tend to use less effective emotion regulation strategies in stressful situations than their noninstitutionalized peers [8,9]. Consistent with this pattern, several meta-analyses have confirmed that emotion regulation mediates the relationship between adverse childhood experiences and increased vulnerability to psychopathology [10,11].

Given that early-life stressors can influence brain–body communication, recent research has examined the associations between interoception and emotional dysregulation [12]. Interoception is the process by which the nervous system detects, interprets, and integrates signals originating from within the body, facilitating a continuous mapping of internal states at both conscious and unconscious levels [13,14]. Existing literature primarily examines one of three key dimensions—interoceptive accuracy, sensitivity, or awareness [15,16]. Among these dimensions, interoceptive accuracy has garnered the greatest empirical attention and is considered a foundational construct that underpins other interoceptive metrics [16]. In this context, studies indicate that childhood trauma experienced in adulthood—including physical, emotional, and sexual abuse, as well as emotional and physical neglect—significantly affects interoceptive accuracy following acute stress exposure, with higher levels of reported trauma associated with greater difficulty in perceiving heartbeats [17]. Similarly, body dissociation, a measure of interoceptive sensitivity, is correlated with childhood traumatic experiences and emotional dysregulation in adulthood and acts as a mediator between these variables [18,19].

Despite increasing interest in interoception, its development, associated factors, and underlying mechanisms in younger populations remain poorly understood. Neurobiological studies have shown that, in six-year-old children, brain regions such as the left insula, cuneus, inferior parietal lobule, and prefrontal cortex are activated during heartbeat detection tasks, resembling adult neural patterns [20]. Interoceptive accuracy has also been positively associated with the amplitude of the heartbeat-evoked potential in both six-month-old infants [21] and adolescents [22]. Evidence further indicates that interoceptive abilities develop over time, particularly through improvements in interoceptive accuracy [23,24].

In adult populations, interoceptive processes have also been linked to prosocial functioning. Positive correlations between interoception and prosocial behavior have been reported [25,26], although causal relationships have not been established. However, empirical data remain sparse regarding the links among interoception, emotion regulation, and prosociality among preschool children. In child populations, these processes have rarely been examined simultaneously, and existing research has focused primarily on isolated aspects of social and emotional functioning [27,28,29]. This gap is particularly relevant, as early childhood represents a critical period for the acquisition of socioemotional competencies and adaptation to environmental demands. It is particularly salient in vulnerable populations exposed to adverse experiences that may disrupt brain–body communication [17,30], as well as in children at risk of cognitive and social impairments, where a more nuanced understanding may inform preventive strategies and facilitate adaptation to new environments [31]. However, this gap persists partly because of unresolved methodological challenges. Addressing this challenge requires the development of paradigms that facilitate the study of interoception in infants and preschool-aged children, particularly tasks that do not depend on advanced comprehension or verbal reporting abilities [24,29]. This constraint is especially critical because existing paradigms may be influenced by prior beliefs or knowledge and may yield subjective responses, as noted for methods such as the heartbeat tracking task (HTT) and the heartbeat discrimination task (HDT) [15,17,32,33,34,35,36,37,38]. The HTT requires participants to report the number of perceived heartbeats over short periods, whereas the HDT involves judging the synchrony between heartbeats and external stimuli [15]. To a lesser extent, interoception has also been examined through sensitivity, typically via self-report measures, and awareness, reflecting the correspondence between confidence in interoceptive ability and actual performance [15,16,39]. Addressing these challenges requires approaches that reduce reliance on advanced cognitive skills, particularly those related to awareness and language [40].

Accordingly, the present study investigates the impact of institutionalization on interoception, emotion regulation, and prosocial behavior in institutionalized preschool children (IPC) and noninstitutionalized preschool children (NIPC), employing age-appropriate paradigms to assess these processes in early childhood.

## 2. Materials and Methods

### 2.1. Participants

A total of 51 preschool children aged between 48 and 72 months (M_age = 66.27, SD_age = 9.55), including 30 boys and 21 girls, participated in the study. The participants were divided into two groups: (1) IPC and (2) NIPC, who served as the control group. The IPC group consisted of 26 preschool children (M_age = 66.22, SD = 10.12) placed in a temporary foster home under the Instituto Colombiano de Bienestar Familiar in Cali, Colombia. At the time of assessment, all the children were undergoing administrative processes aimed at restoring their rights. The NIPC group consisted of 25 noninstitutionalized preschool children (M_age = 66.31, SD = 9.12) recruited from early childhood education centers in Cali, Colombia. These children had lived with their biological families since birth and had no history of institutionalization. Only typically developing children were included in both groups. The inclusion criterion was that the parents of the participants had no history of neurological or systemic diseases, psychiatric or developmental disorders, or intellectual disability. This was determined based on clinical history and evaluations conducted by the medical and psychosocial team of the institution for the IPC group and parental self-reports for the NIPC group. Participants from both groups were matched for age, sex, and socioeconomic status. Legal guardians were contacted through the institutions where the children participated and were informed about the research; written informed consent was obtained from them before the evaluation. In addition, verbal assent was obtained from each child before the tasks began. The study was conducted in accordance with the Declaration of Helsinki and was approved by the Human Research Ethics Committee of Universidad Icesi (Approval Act #591/2024).

### 2.2. Tasks

#### 2.2.1. Interoception

**Figure 1:**
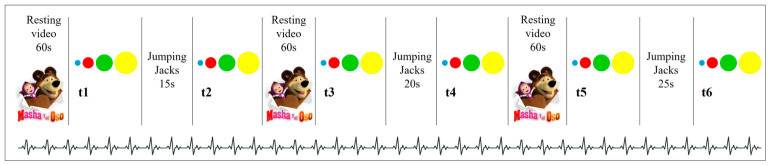
Schematic overview of the Jumping Jack Paradigm (JJP). Heart rate was recorded continuously while children provided self-reported estimates of their heart rate at six time points (t1–t6). Assessments were conducted before and after three jumping-jack exercise blocks lasting 15 s, 20 s, and 25 s, allowing comparison between objective cardiac activity and subjective heart-rate perception across varying levels of physical exertion.

The tasks were designed as simple games that posed no physical, cognitive, or emotional risk to the participants. Two tasks were implemented: one assessed interoceptive accuracy, and the other assessed interoceptive sensitivity. An adapted version of the Jumping Jack Paradigm (JJP), previously employed in studies assessing interoceptive accuracy in preschool children aged 4–6 years, was used in the present study [38,41]. This task involved heartbeat tracking after participants performed jumping jacks across three time intervals (15, 20, and 25 s) to facilitate the perception of internal bodily states. Given the arithmetic abilities typical of this age group, children estimated their heart rate by selecting one of four circles, each varying in size and color, with larger circles representing a higher heart rate. To support understanding, each circle was paired with a color and an animal representing a distinct speed level: (1) blue, very slow, turtle; (2) red, slow, penguin; (3) green, fast, cat; and (4) yellow, very fast, jaguar. This visual and symbolic coding helped strengthen the association between perceived internal rhythm and external stimuli in a playful and accessible way (Figure 1).

The task was developed in Python 3.10, using libraries such as Pygame to build an interactive visual interface and Bleak for Bluetooth communication with the Polar H10 heart rate monitor. The Polar H10 heart rate monitor is a portable device that provides electrocardiogram-quality data and poses no risk to users’ well-being, as it does not cause discomfort or produce adverse physical, cognitive, or emotional effects. It has been widely used in pediatric populations, including children aged 7–16 years, with no negative events reported to date [42,43]. Therefore, it was used to obtain objective measures of heart rate variability, which were compared to the children’s subjective responses.

An official Polar Software 6.3.0 Development Kit (SDK) was used to manage device connections, enabling real-time reception and processing of physiological data. Additionally, multithreading was implemented to run the experimental protocol and physiological recording simultaneously, ensuring accurate synchronization between task events and heart rate measurements. Data were automatically stored in .xlsx files, including continuous heart rate recordings and event-related tags for each phase of the protocol (e.g., “start_video,” “start_exercise,” “end_exercise,” and “rest_post_exercise). This structure enabled subsequent data segmentation based on key experimental events and supported both temporal and comparative analyses.

**Figure 2:**
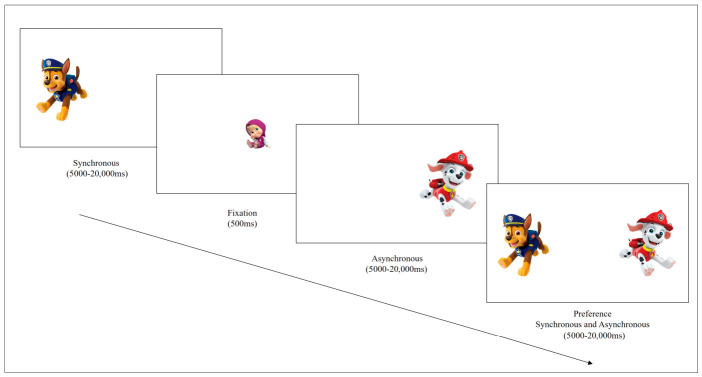
Schematic overview of the iBEAT task. Children discriminated between heartbeat-synchronous and heartbeat-asynchronous stimuli in a sequential gaze paradigm. A total of 60 individual trials were presented, with synchronous and asynchronous stimuli counterbalanced across animated characters (Paw Patrol characters #1 and #2) and screen position (left/right). A central distractor was displayed for 500 ms before each trial to orient attention. Stimuli remained on screen for a minimum of 5 s and up to 20 s, depending on the child’s gaze behavior. Trials ended when the child looked away for more than 2 s. If more than four consecutive trials lasted less than the minimum viewing duration, the individual-trial phase was terminated and both stimuli were presented simultaneously to assess preference.

To assess interoceptive sensitivity, the iBEAT task was used. This task employs a sequential visual paradigm to evaluate children’s ability to discriminate between synchronous and asynchronous stimuli relative to their heartbeat and to assess their final preference for either stimulus. Each trial presented a visual stimulus that moved in synchrony or asynchrony with the child’s heartbeat, which was monitored in real time using the Polar H10 heart rate monitor. Continuous eye movements were monitored using a Tobii eye tracker with the Pro SDK, operating at a sampling rate of 120 Hz and positioned above the computer screen. This task has been replicated in various studies involving infants under 1 year of age, demonstrating its safety—posing no physical, cognitive, or emotional risk—and its validity as a measure of the construct [21,27,37,44]. Given that the present study involved preschool children aged 4–6 years, the stimuli were adapted to be more engaging for this age group by incorporating animated Paw Patrol characters. The task consisted of 60 individual trials and 8 preference trials, with a maximum duration of 23 min. The stimuli were counterbalanced by animated characters (Paw Patrol characters #1 and #2) and screen position (left–right) to avoid biased outcomes. Before each stimulus, a fixation figure was presented at the center of the screen for 500 ms to maintain the child’s attention on the display. Each stimulus was initially displayed for a minimum of 5000 ms. The trial duration then depended on the child’s visual attention, with a maximum duration of 20,000 ms. If the child stopped looking at the stimulus for more than 2000 ms, the trial ended, and the next one began. If four consecutive trials lasted less than 5000 ms, the phase of individual stimulus presentation was concluded, and both stimuli were presented simultaneously to assess preference (Figure 2).

From a technical perspective, the task was programmed in Python, integrating real-time heart rate monitoring and eye tracking. The Bleak library and Polar SDK were used for Bluetooth communication with the Polar H10 heart rate monitor, and the official Tobii SDK was employed to capture continuous eye movement data. Multithreaded and asynchronous programming ensured smooth task execution and precise synchronization among visual, physiological, and behavioral events. The interface was developed using Pygame, and stimulus movement was dynamically adjusted in real time based on heart rate data from the Polar H10, enabling controlled synchronous and asynchronous conditions relative to the participant’s heartbeat. Eye tracking was conducted using a Tobii device connected via USB, with its proprietary software and calibration tool ensuring accuracy before each session. During task execution, data from each trial, including the experimental condition, gaze coordinates, presented stimulus, and heart rate, were automatically stored in .xlsx files, allowing for precise data segmentation for later analysis.

#### 2.2.2. Prosocial Behavior: Cooperation

The fishing task assesses prosocial behavior through cooperation in 4-year-old children [45]. The original version of the task was modified to make it more engaging for children aged 4–6 years by using rubber bath animals instead of colored plastic clips. Twenty rubber animals were placed in a plastic container, and both the child and the experimenter were given fishing rods connected to facilitate coordinated action. The goal of the game was to “catch” as many animals as possible together within 3 min.

#### 2.2.3. Emotion Regulation—Inhibitory Control

The delayed gratification choice task has been used and adapted in several studies to assess the ability of preschool-aged children (3–5 years old) to delay gratification and thereby demonstrate inhibitory control [46,47,48,49]. The version implemented in this study followed adaptations proposed in previous work [50,51]. Children were presented with three sticker themes and offered a choice between receiving an envelope containing one sticker from their favorite theme immediately or waiting until later to receive an envelope containing three stickers from that same theme.

### 2.3. Questionnaires

#### Emotion Regulation

The Early Emotion Regulation Behavior Questionnaire (EERBQ) was completed by primary caregivers to assess behavioral emotion regulation strategies in children aged 2–6 years across both positive and negative emotional contexts. Its psychometric properties have been evaluated, and the results provide initial support for the internal consistency and construct validity of the battery as a measure of behavioral emotion regulation strategies in young children [52].

The questionnaire includes 12 hypothetical scenarios representing common emotionally evocative events: three eliciting anger, three eliciting fear, three eliciting sadness, and three eliciting excitement. For each scenario, caregivers rated the likelihood that the child would engage in each of eight behavioral regulation strategies (mindfulness, avoidance, distraction, verbal help-seeking, physical help-seeking, self-soothing, verbal venting, or physical venting) using a 7-point Likert scale ranging from 1 (not at all likely) to 7 (very likely). The final section of the questionnaire measures negative emotional reactivity. The caregivers rated six items, including “my child has strong emotional reactions” and “I find it easy to get my child to calm down,” using a 7-point Likert scale ranging from 1 (strongly disagree) to 7 (strongly agree).

### 2.4. Procedure

**Figure 3:**
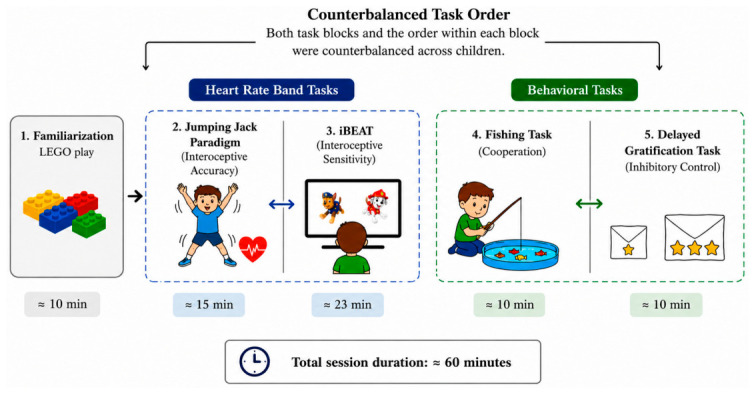
Experimental procedure and counterbalancing design. Task order was counterbalanced across participants. The JJP and iBEAT tasks were always administered consecutively because both required heart-rate monitoring. Likewise, the Fishing Task and Delayed Gratification Task were administered consecutively as a behavioral block. Total session duration was approximately 60 min.

The protocol began with a familiarization phase consisting of approximately 10 min of playing with LEGO blocks with the child. After this time, the caregiver present in the room was asked to place the heart rate monitor band on their own body to show both themselves and the child how their heartbeat appeared on the mobile phone screen. This demonstration aimed to build trust with the child and motivate them to allow the band to be placed on their own body. Although the children did not place the band themselves, they were encouraged to engage actively and allow it to be placed. Once the child agreed, the experimenter continued playing with them for a few additional minutes to help them become accustomed to wearing the device. After the familiarization phase, the toys were removed, and the caregiver was given instructions on how to behave during the protocol to avoid interrupting or influencing the process. The four experimental tasks were counterbalanced, and the questionnaire was administered to the primary caregiver at the end of the session. The sequence of the task procedures is presented in Figure 3.

Before beginning the JJP, the child was taught the meaning of the circle scale used in the Jumping Jack task to evaluate how fast their heart was beating. The protocol continued only after the child demonstrated a clear understanding and memorization of the four circles, each representing a heartbeat speed associated with a different animal. The JJP began with a resting period during which the child was asked to watch a 60-s video featuring Masha and the Bear, one of the most popular cartoons among children in this age group.

After the video, the child was asked to select one of the four circles displayed on the screen to answer the question “How fast is your heart beating?” Subjective self-reported and objective heart rate data were recorded after each resting period (t1, t3, and t5) and again 30 s after each of the three Jumping Jack blocks, which progressively increased in duration (t2—15 s, t4—20 s, and t6—25 s). After stating their circle selection aloud, the child was instructed to immediately stand up and perform jumping jacks in synchrony with the experimenter, who also performed the movements. The full paradigm lasted approximately 15 min (Figure 1).

For the iBEAT task, the child was asked to sit in front of the computer, complete the calibration procedure, and maintain visual fixation on the screen while stimuli were presented.

For the fishing task, the objective of the game was explained, along with the rule that each participant could choose which animal to catch on each turn. Each child received verbal and visual instructions on how to handle the “fishing rods” without touching the line or hook and how to collaboratively catch the animals. A familiarization phase was conducted first, and the task began only after the child successfully caught at least one animal in cooperation with the experimenter. The game lasted 3 min, during which the experimenter used encouraging phrases to reinforce cooperation, such as “we’re doing a great job” and “we can do it”.

For the delayed gratification choice task, the child was shown three sticker sets with different themes (Paw Patrol, Masha and the Bear, and Animals), each containing 15 stickers, and was asked to select their favorite. The child was also invited to choose one of three colored cups (gold, pink, or silver) to use during the game. After the selections were made, two envelopes were placed in front of the child: one marked with a single red circle and the other with three red circles. The child was then given an explanation of the meaning of each envelope and the implications of choosing one over the other. It was explained that choosing the envelope with one red circle would result in receiving one sticker from their favorite set immediately, which they could place on their chosen cup, whereas selecting the envelope with three red circles would result in receiving three stickers from the same set, but only after a delay. Once comprehension was confirmed, the child was asked across four separate trials which sticker set they wanted to play with and which envelope they preferred. After completing these tasks, the child was invited to return to playing with the LEGO blocks while the experimenter administered the questionnaire to the primary caregiver.

### 2.5. Data Analysis

Interoceptive accuracy (IAc) was calculated by first obtaining three objective and three subjective measurements for each participant before and after the exercise. Objective measurements reflected the average heart rate during the 5 s immediately preceding the participant’s subjective report, i.e., just before pressing the circle selection button. The three pre-exercise and three post-exercise values were averaged separately for both objective and subjective measures. Change scores were then computed by subtracting the pre-exercise mean from the post-exercise mean for each measure. After these scores were standardized using z scores, IAc was calculated as the difference between the standardized subjective and objective change scores. Values closer to 0 indicate smaller discrepancies between subjective responses and objective recordings. A positive score indicates that the change in the subjective response was greater than that in the objective recording. In contrast, a negative score indicates that the change in the objective recording was greater than that in the subjective response [38].

To assess interoceptive sensitivity, the average gaze duration toward asynchronous and synchronous options during the last eight stimuli of the iBeat task was used to determine stimulus preference based on viewing time. Sensitivity was estimated as the difference in average fixation times between asynchronous and synchronous stimuli.

Cooperation data were obtained by averaging the number of successfully caught animals during the task. For inhibitory control, a score of 0 was assigned when the child chose one sticker, and a score of 1 was assigned when the child chose three stickers. The total score was calculated as the average score across the four trials. The EERBQ yielded eight subscales that reflect qualitatively distinct behavioral responses to both positive and negative emotions. Each subscale was calculated as the mean score of the 12 items corresponding to each emotion regulation strategy. Additionally, emotional reactivity was computed by averaging the final six questionnaire items.

Finally, group differences between institutionalized (IPC) and noninstitutionalized preschool children (NIPC) were examined using two separate one-way multivariate analyses of variance (MANOVAs), with institutionalization as the between-group factor. The first MANOVA included task-based measures of interoception (accuracy and sensitivity), cooperation, and inhibitory control (Table 1), whereas the second included emotion regulation dimensions derived from the EERBQ (Table 2). Additionally, multiple linear regression analyses were performed to examine the relationships between institutionalization, interoception, emotion regulation, and cooperation outcomes across all participants. Further regression models were constructed incorporating significantly correlated variables as predictors. In these models, IAc and cooperation were entered as dependent variables (Table 3).

## 3. Results

The results are organized into four sections. First, interoception is characterized by IPC and NIPC. Second, prosocial behavior is described through cooperation in IPC and NIPC. Third, emotion regulation levels are examined across groups. Finally, the associations among interoception, emotion regulation, and prosocial behavior are explored through cooperation. Interoceptive sensitivity scores reported in Table 1 correspond to the difference in fixation time between asynchronous and synchronous stimuli, with positive values indicating a greater preference for asynchronous stimuli.

Significant multivariate effects of institutionalization were observed for both task-based measures (Pillai’s Trace = 0.231, F(4, 46) = 3.45, *p* = 0.015) and parent-reported emotion regulation dimensions (Pillai’s Trace = 0.600, F(9, 41) = 6.83, *p* < 0.001). Follow-up univariate analyses indicated group differences in interoceptive accuracy, cooperation, avoidance, physical help-seeking, self-soothing, verbal venting, and physical venting, whereas no significant differences were found for interoceptive sensitivity, inhibitory control, emotional reactivity, mindfulness, distraction, or verbal help-seeking. Descriptive statistics, multivariate test results, and follow-up univariate analyses are presented in Table 1 and Table 2.

### 3.1. Institutionalization and Interoception

Interoception was characterized in both IPC and NIPC, considering IAc and interoceptive sensitivity (Table 1).

Both groups showed mean IAc values close to zero, indicating relatively small discrepancies between subjective and objective changes in heart rate at the group level. However, despite these small overall discrepancies, the direction of the estimation error differed significantly between groups. The NIPC group showed a positive mean score, suggesting that, on average, these children overestimated changes in their heart rate. Specifically, 68% of noninstitutionalized children scored above zero, indicating that they perceived greater heartbeat changes than actually occurred. In contrast, the IPC group presented a negative mean score, indicating that, on average, children underestimated the changes in their heart rate. In particular, 62% of the institutionalized children scored below zero, suggesting that they perceived smaller changes than those recorded.

Regarding interoceptive sensitivity, IPC showed an average fixation time of 5526.86 ms (SD = 2295.29) for synchronous stimuli and 6036.01 ms (SD = 2630.10) for asynchronous stimuli. Similarly, NIPC showed average fixation times of 6030.65 ms (SD = 1547.51) for synchronous stimuli and 6562.76 ms (SD = 2291.21) for asynchronous stimuli. Within-group comparisons revealed no significant differences between synchronous and asynchronous fixation times (IPC: *p* = 0.354; NIPC: *p* = 0.310). Likewise, between-group comparisons were not significant for either synchronous stimuli, F(1,49) = 0.84, *p* = 0.365, or asynchronous stimuli, F(1,49) = 0.58, *p* = 0.450. Descriptively, both groups exhibited longer fixation durations for asynchronous than synchronous stimuli.

To formally evaluate discrimination between synchronous and asynchronous stimuli, a mixed ANOVA was conducted with group (IPC vs. NIPC) as a between-subject factor and condition (synchronous vs. asynchronous) as a within-subject factor to explicitly test discrimination ability. The analysis revealed no significant main effects or interactions, indicating that children did not reliably differentiate between synchronous and asynchronous stimuli (group: F(1,49) = 1.05, *p* = 0.310, ges = 0.014; condition: F(1,49) = 1.96, *p* = 0.168, ges = 0.014; group × condition: F(1,49) < 0.01, *p* = 0.976, ges < 0.001).

### 3.2. Institutionalization and Cooperation

Compared with the IPC group, the NIPC group exhibited significantly higher levels of cooperation (Table 1).

### 3.3. Institutionalization and Emotion Regulation

As shown in Table 1 and Table 2, no statistically significant differences were found between the two groups in inhibitory control. However, there were significant differences in several emotion regulation strategies, specifically in the dimensions of avoidance, physical help-seeking, self-soothing, and verbal and physical venting.

### 3.4. Institutionalization, Interoception, Emotion Regulation, and Cooperation

Multiple regression analyses were conducted to examine relationships among interoception, emotion regulation, and prosocial behavior in the IPC and NIPC groups. As presented in Table 3, institutionalization and interoceptive sensitivity significantly predicted IAc. Additionally, emotion regulation strategies, specifically avoidance, physical venting, verbal venting, and self-soothing, along with synchronous preference, significantly predicted cooperative behavior.

## 4. Discussion

This study provides new evidence that early institutionalization is associated with alterations in the processing of internal bodily signals, emotion regulation, and prosocial behavior in children. To the best of our knowledge, this is the first study to simultaneously examine interoception, emotion regulation, and cooperation among institutionalized and noninstitutionalized preschool-aged children. In this context, early adversities may disrupt the connection between bodily signals and cognition, attenuating the link between internal and external experiences [53]. In line with previous studies using the same paradigm to assess interoceptive accuracy [38,41], children’s ability to consciously detect internal bodily changes varies when they become physically aroused. Our results revealed a difference in IAc between the groups. Although both groups had mean values close to zero, consistent with evidence suggesting that most children estimate their heart rate approximately as expected [38], the direction of the estimation bias differed between groups. Institutionalized children showed a mean IAc below zero, indicating a tendency to underestimate their heart rate. In contrast, noninstitutionalized children had a mean IAc above zero, suggesting a tendency toward overestimation. This discrepancy may be attributed to early exposure to stressors, such as psychosocial deprivation [54], which can disrupt communication between the brain and body [17]. These patterns may contribute to differences in internal bodily signals due to chronic dysregulation, leading to a persistently altered perception of bodily sensations [11]. This finding is consistent with research suggesting that early adversity impairs the interpretation of bodily signals, resulting in altered physiological processing [55].

Conversely, the propensity to overestimate internal signals among noninstitutionalized children may be attributed to the emotional amplification within their familial environments, which promote closeness and satisfaction, even when these emotions are not authentically experienced [56]. The observed patterns suggest that interoceptive biases may manifest in different directions, depending on familial and social contexts [57]. Consequently, children may internalize these exaggerated responses, perceive their own bodily signals as more intense than they actually are, and overestimate certain physiological reactions, even when they are absent. This pattern might be linked to confirmation bias, which involves seeking physical signals to confirm expectations, even when those expectations are inaccurate [58], such as the belief that physical activity invariably results in a significant increase in heart rate.

In terms of interoceptive sensitivity, both institutionalized and noninstitutionalized children were unable to distinguish between synchronous and asynchronous stimuli, although both groups showed prolonged fixation durations on asynchronous stimuli. Although no statistically significant differences were identified between the groups, noninstitutionalized children exhibited greater mean fixation times on synchronous stimuli than their institutionalized peers. This observation is consistent with the possibility that early adverse experiences and institutional environments may influence these skills [11,17,54]. These findings align with previous research on infants aged 5–6 months, which reported partial evidence of a preference for asynchronous stimuli and the ability to differentiate between the two types [21,37,40,59]. In this study, the results suggest that interoceptive sensitivity and institutionalization are significant, albeit not exclusive, predictors of IAc. While both variables were predictive, children with heightened sensitivity to internal signals tended to detect bodily changes more readily; however, their ability to accurately interpret these changes did not necessarily increase, particularly when accuracy exceeded the zero threshold.

Differences in cooperation were observed between the two groups. Noninstitutionalized children had notably higher scores than institutionalized children, corroborating previous research that has associated institutionalization with dysregulation of social skills during childhood and adolescence [54,60,61,62]. The findings indicate a potential association between social behavior and embodied processes that integrate interoception and emotion regulation. In the domain of emotion regulation, substantial differences were observed in avoidance, physical help seeking, self-soothing, and verbal and physical venting. Furthermore, the findings indicated that the dimensions of emotion regulation—specifically, avoidance, verbal and physical venting, and self-regulation—as well as the capacity to detect synchronization with one’s own heartbeat, significantly predicted cooperation. These findings suggest that social functioning depends on the interplay between bodily perception and emotion regulation. These results are consistent with studies indicating that synchronous actions between individuals significantly affect prosocial behavior, perceived social bonds, social cognition, and positive affect [63] and that emotional dysregulation is linked to challenges in prosocial behavior [64]. Regarding emotion regulation strategies, institutionalized children scored significantly higher on rudimentary strategies, such as physical and verbal venting, self-soothing, and avoidance. These strategies, which are often considered maladaptive, can reduce emotional intensity with minimal cognitive effort but involve greater social challenges. In contrast, more sophisticated strategies require greater cognitive control but promote more adaptive social interactions [52]. Consistent with previous evidence, children from institutional environments show difficulties in emotion regulation [1]. Adopted children from such contexts have less developed emotion regulation skills or are less efficient in using them in stressful play situations, often constructing narratives characterized by aggressive content, anxiety, insecure attachment, and conflict avoidance [8]. This pattern is consistent with research showing that cumulative childhood trauma predicts increased reliance on maladaptive emotion regulation strategies and decreased use of adaptive strategies [7].

In contrast, it is important to highlight that the institution involved in this study offers a high standard of care, likely corresponding to levels 3 and 4 in Gunnar’s taxonomy. Specifically, level 3 institutions meet all the needs of children except those related to establishing stable and long-term relationships with consistent caregivers. Level 4 institutions address these challenges more extensively, but still do not provide a regular family life in a typical social environment [65]. Evidence shows that interventions aimed at increasing stability and consistency in caregiving, as well as promoting sensitive and responsive caregiver–child interactions, result in the greatest improvements in child development across both physical and behavioral domains [66]. Similarly, institutional care can provide a safe, supportive environment that promotes resilience in children [67,68]. Moreover, resilience is not only an individual trait but also a dynamic process shaped by the child’s ability to negotiate with their environment to access meaningful resources. In this sense, the extent to which the environment facilitates access to these resources shapes the child’s developmental trajectory, with developmental outcomes varying according to the level of environmental support available [69]. This perspective may help explain the high scores in adaptive strategies and the possible lack of statistically significant differences between groups in these dimensions.

### Limitations and Future Research

This study has several limitations that should be considered in future research. Future studies should aim to increase sample size to facilitate more comprehensive analyses by age, sex, and other variables. It is also important to consider including an additional group of noninstitutionalized children in vulnerable conditions. Regarding the tasks used to assess interoception, the JJP, previously used with preschool children, proved to be methodologically suitable for evaluating interoceptive accuracy. Nevertheless, exploring alternatives that minimize the influence of prior beliefs or random responses is recommended to obtain more precise measurements. Although performing jumping jacks increased the children’s heart rate by an average of 14 beats per minute, this change might not have been sufficient for them to consciously detect and report as a subjective response. Future studies should consider increasing the duration of activities used to induce greater physiological activation, thereby enhancing children’s interoceptive perception. Furthermore, although the heartbeat counting task is widely used to assess interoceptive accuracy, the potential influence of perceptual factors on the results cannot be ruled out.

Additionally, response scales should include the option “I feel nothing” to reduce the likelihood of random responses [41]. Furthermore, the task used to assess interoceptive sensitivity had not been previously applied to preschool children but only to infants aged 5–6 months. Although the stimuli were adapted to the participants’ age, the task proved complex and required significant attentional resources. Future research should explore alternative tasks that allow continuous subjective assessment of this aspect of interoception or modify the current task by reducing the habituation period to ensure that stimulus preference is captured before attentional fatigue occurs.

## 5. Conclusions

This study offers novel insights into the complex interplay among interoception, emotion regulation, and cooperation in institutionalized and noninstitutionalized preschool children. As the first study to jointly evaluate these variables in this population, it provides valuable information that may serve as a foundation for future interventions in institutional care settings. The findings highlight that early-life experiences related to institutionalization and variations in the quality and stability of caregiving environments are related to children’s ability to perceive internal bodily signals, regulate emotions, and engage in prosocial behaviors. Although the institutionalized children demonstrated difficulties in interoceptive accuracy and certain rudimentary regulation strategies, they also demonstrated high levels of adaptive emotion regulation, suggesting the presence of resilience processes. Importantly, the high standard of care provided by the institution involved may have contributed to these adaptive outcomes. This underscores the relevance of viewing resilience not as a fixed individual trait but as a dynamic process shaped by the availability of meaningful resources within the child’s environment. This study advances the understanding of how early adversity and protective caregiving environments interact to influence socioemotional and interoceptive development. Future research is needed to deepen the understanding of these phenomena, explore their multiple dimensions, and refine the methods appropriate for early childhood populations. From a clinical perspective, these findings underscore the importance of considering interoception and emotion regulation as potentially relevant targets for developing intervention and prevention strategies for children exposed to early adversity.

## Figures and Tables

**Table 1:** MANOVA of Task Measures With Group Means and Univariate Follow-Up Tests.

Measure	IPC Group Mean (SD)	NIPC Group Mean (SD)	F (df)	*p*	η^2^p
Multivariate (Pillai’s trace)	—	—	3.45 (4, 46)	0.015 *	0.231
IAC	−0.23 (1.02)	0.49 (1.20)	5.29 (1, 49)	0.026 *	0.097
Interoceptive sensitivity	509.15 (2746.64)	532.11 (2563.85)	0.00 (1, 49)	0.976	0.000
Cooperation	3.92 (2.33)	5.60 (2.18)	7.03 (1, 49)	0.011 *	0.125
Inhibitory control	0.53 (0.40)	0.41 (0.38)	1.17 (1, 49)	0.284	0.023

Note. Pillai’s trace V = 0.231. IPC = institutionalized preschool children; NIPC = non-institutionalized preschool children. Multivariate MANOVA (Pillai’s trace) followed by univariate tests for each dependent variable. Group means and standard deviations are shown for each measure. η^2^p = partial eta-squared; multivariate η^2^p is based on Pillai’s trace. N = 51. * *p* < 0.05.

**Table 2:** MANOVA of Emotion Regulation Scales With Group Means and Univariate Follow-Up Tests.

Measure	IPC Group Mean (SD)	NIPC Group Mean (SD)	F (df)	*p*	η^2^p
Multivariate (Pillai’s trace)	—	—	6.83 (9, 41)	<0.001 ***	0.600
Emotional reactivity	3.33 (0.93)	3.49 (0.74)	0.46 (1, 49)	0.502	0.009
Mindfulness	4.92 (1.46)	5.10 (1.17)	0.24 (1, 49)	0.630	0.005
Avoidance	3.46 (1.21)	2.28 (1.03)	13.94 (1, 49)	<0.001 ***	0.221
Distraction	4.81 (0.89)	5.17 (0.86)	2.05 (1, 49)	0.158	0.040
Verbal help-seeking	5.65 (0.76)	5.53 (0.99)	0.25 (1, 49)	0.617	0.005
Physical help-seeking	5.58 (0.85)	4.91 (1.13)	5.76 (1, 49)	0.020 *	0.105
Self-soothing	2.62 (1.33)	1.74 (0.99)	7.26 (1, 49)	0.010 **	0.129
Verbal venting	3.72 (1.47)	2.85 (1.21)	5.29 (1, 49)	0.026 *	0.097
Physical venting	3.52 (1.50)	2.19 (0.58)	17.18 (1, 49)	<0.001 ***	0.260

Note. Pillai’s trace V = 0.600. Parent-reported emotion regulation scales in the order shown. Multivariate MANOVA (Pillai’s trace) followed by univariate tests for each scale. IPC = institutionalized; NIPC = non-institutionalized. η^2^p = partial eta-squared; multivariate η^2^p is based on Pillai’s trace. N = 51. * *p* < 0.05, ** *p* < 0.01, *** *p* < 0.001.

**Table 3:** Regression coefficients for the IAc and cooperation models.

Dependent Variable	Model	F	Df	Adjusted R^2^	Predictor	Β	SE	*t*	*p*
IAC	1	5.15	2.48	0.14	Intercept		0.47	−1.87	0.067
					Institutionalization	0.31	0.30	2.39	0.021 *
					Interoceptive sensitivity	−0.28	0.00	−2.15	0.037 *
Cooperation	2	16.44	2.48	0.38	Intercept		1.03	5.25	<0.001 ***
					Synchronous preference	0.33	0.00	2.98	0.00 **
					Avoidance	−0.54	0.21	−4.89	<0.001 ***
Cooperation	3	12.27	2.48	0.31	Intercept		1.08	4.63	<0.001 ***
					Synchronous preference	0.31	0.00	2.67	0.010 *
					Physical venting	−0.48	0.21	−4.06	<0.001 ***
Cooperation	4	10.48	2.48	0.28	Intercept		1.12	4.34	<0.001 ***
					Synchronous preference	0.34	0.00	2.79	0.01 *
					Verbal venting	−0.44	0.21	−3.65	<0.001 ***
Cooperation	5	8.28	2.48	0.23	Intercept		1.10	3.85	<0.001 ***
					Synchronous preference	0.30	0.00	2.41	0.020 *
					Self-soothing	−0.38	0.24	−3.07	0.004 **

Note. N = 51. Institutionalization (Institutionalized Preschool Children and Non-Institutionalized Preschool Children); F, F-statistic; df, degrees of freedom; adjusted R^2^, adjusted R-squared; β, standardized regression coefficient; SE, standard error; *t*, t-statistic; *p*, *p* value. * *p* < 0.05, ** *p* < 0.01, *** *p* < 0.001. Model 1 examines whether interoceptive accuracy (IAc) is predicted by group type (institutionalization) and interoceptive sensitivity. Models 2, 3, 4, and 5 examine whether cooperation is predicted by synchronous preference and different emotion regulation strategies (avoidance, physical venting, verbal venting, and self-soothing).

## Data Availability

The data presented in this study are available on request from the corresponding author due to privacy restrictions.
